# *N*,*N*′-Bis(4-chloro­phen­yl)naphthalene-1,4-dicarboxamide *N*,*N*-dimethyl­formamide disolvate

**DOI:** 10.1107/S1600536809034394

**Published:** 2009-09-09

**Authors:** Lin-Hai Jing

**Affiliations:** a School of Chemistry and Chemical Engineering, China West Normal University, Nanchong 637002, People’s Republic of China

## Abstract

In the title compound, C_24_H_16_Cl_2_N_2_O_2_·2C_3_H_7_NO, the two C=O groups adopt an *anti* orientation. The two amide groups are twisted away from the naphthalene ring system by 59.10 (4) and 68.22 (4)°. The crystal packing is stabilized by N—H⋯O and C—H⋯O hydrogen bonds.

## Related literature

For the use of 1,4-naphthalene­dicarboxylic acid derivatives in the preparation of polymers, see: Fukuzumi *et al.* (1994 ▶); Tsukada *et al.* (1994 ▶). For related structures, see: Jing (2008 ▶); Jing *et al.* (2006*a* ▶,*b* ▶).
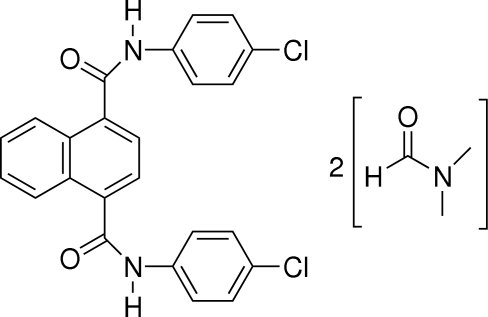


## Experimental

### 

#### Crystal data

C_24_H_16_Cl_2_N_2_O_2_·2C_3_H_7_NO
*M* *_r_* = 581.48
Triclinic, 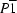

*a* = 12.040 (3) Å
*b* = 12.121 (3) Å
*c* = 12.295 (3) Å
α = 101.75°
β = 111.843 (3)°
γ = 110.833 (2)°
*V* = 1435.1 (6) Å^3^
*Z* = 2
Mo *K*α radiation
μ = 0.27 mm^−1^
*T* = 93 K
0.40 × 0.33 × 0.30 mm

#### Data collection

Rigaku SPIDER diffractometer
Absorption correction: none
11770 measured reflections
6317 independent reflections
5120 reflections with *I* > 2σ(*I*)
*R*_int_ = 0.021

#### Refinement

*R*[*F* ^2^ > 2σ(*F* ^2^)] = 0.036
*wR*(*F* ^2^) = 0.095
*S* = 1.00
6317 reflections
373 parameters
H atoms treated by a mixture of independent and constrained refinement
Δρ_max_ = 0.35 e Å^−3^
Δρ_min_ = −0.22 e Å^−3^

### 

Data collection: *RAPID-AUTO* (Rigaku, 2004 ▶); cell refinement: *RAPID-AUTO*; data reduction: *RAPID-AUTO*; program(s) used to solve structure: *SHELXS97* (Sheldrick, 2008 ▶); program(s) used to refine structure: *SHELXL97* (Sheldrick, 2008 ▶); molecular graphics: *XP* in *SHELXTL* (Sheldrick, 2008 ▶); software used to prepare material for publication: *SHELXL97*.

## Supplementary Material

Crystal structure: contains datablocks global, I. DOI: 10.1107/S1600536809034394/ci2896sup1.cif

Structure factors: contains datablocks I. DOI: 10.1107/S1600536809034394/ci2896Isup2.hkl

Additional supplementary materials:  crystallographic information; 3D view; checkCIF report

## Figures and Tables

**Table 1:** Hydrogen-bond geometry (Å, °)

*D*—H⋯*A*	*D*—H	H⋯*A*	*D*⋯*A*	*D*—H⋯*A*
N1—H1*N*⋯O4^i^	0.85 (2)	2.01 (2)	2.8536 (17)	173 (2)
N2—H2*N*⋯O3	0.89 (2)	2.02 (2)	2.9040 (17)	168 (2)
C23—H23⋯O4	0.95	2.39	3.3258 (19)	167
C2—H2⋯O3^ii^	0.95	2.54	3.4458 (19)	160
C6—H6⋯O2^iii^	0.95	2.38	3.3214 (19)	169
C16—H16⋯O3^i^	0.95	2.54	3.3949 (19)	151
C21—H21⋯O1^iv^	0.95	2.37	3.1719 (19)	142

Symmetry codes: (i) 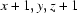; (ii) 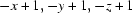; (iii) 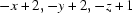; (iv) 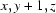.

**Fig. 1.**
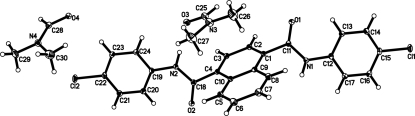
The asymmetric unit of the title compound, showing 30% probability displacement ellipsoids and the atomic numbering.

## supplementary crystallographic information

### Comment

1,4-Naphthalenedicarboxylic acid derivatives are a class of intermediates important for applications as monomers in the preparation of polymers (Fukuzumi *et al.*, 1994; Tsukada *et al.*, 1994). Previously, we have reported crystal structures of *N*,*N*'-bis(4-nitrophenyl)naphthalene-1,4-dicarboxamide dimethylsulfoxide disolvate (Jing *et al.*, 2006*a*), *N*,*N*'-bis(2-methoxyphenyl)naphthalene-1,4-dicarboxamide (Jing *et al.*, 2006*b*) and *N*,*N*'-bis(4-methylphenyl)-1,4-naphthalenedicarboxamide *N*,*N*-dimethylacetamide disolvate (Jing, 2008). We now report the crystal structure of the title compound.

Bond lengths and angles in the molecules are normal. The naphthalene ring system is planar, with a maximum deviation of 0.056 (1) Å for atom C2. Two C═O groups exhibit an anti orientation. As a result of steric effects, the substituent groups at atoms C1 and C4 are twisted away from the plane of the naphthalene ring system(Fig. 1). The O1/N1/C1/C11 and O2/N2/C4/C18 planes form dihedral angles of 59.10 (4) and 68.22 (4)°, respectively, with the C1-C10 plane. The O1/N1/C1/C11 and C12—C17 planes are inclined at an angle of 12.15 (8)° while the O2/N2/C4/C18 and C19—C24 planes make a dihedral angle of 11.22 (9)°. The crystal packing is stabilized by N—H···O and C—H···O hydrogen bonds (Table 1).

### Experimental

Naphthalene-1,4-dicarboxylic acid (2 mmol) and an excess of thionyl chloride (6 mmol) in dioxane (20 ml) were boiled under reflux for 6 h. The solution was distilled under reduced pressure and a yellow solid was obtained. *p*-Chloroaniline (4 mmol) in tetrahydrofuran (20 ml) was added to the yellow solid and boiled under reflux for 6 h. The solution was then cooled to ambient temperature and filtered to remove the tetrahydrofuran. The precipitate obtained was dissolved in dimethylformamide and allowed to stand for one month at ambient temperature, after which time colourless single crystals suitable for X-ray diffraction were obtained.

### Refinement

N-bound H atoms were located in a difference Fourier map and refined isotropically [N-H = 0.849 (18) and 0.895 (19) Å]. C-bound H atoms were placed in calculated positions, with C-H = 0.95 or 0.98 Å, and refined using a riding model, with *U*_iso_(H) = 1.2*U*_eq_(C).

### Crystal data

**Table:**

C_24_H_16_Cl_2_N_2_O_2_·2C_3_H_7_NO	*Z* = 2
*M**_r_* = 581.48	*F*(000) = 608
Triclinic, *P*1	*D*_x_ = 1.346 Mg m^−^^3^
Hall symbol: -P 1	Mo *K*α radiation, λ = 0.71073 Å
*a* = 12.040 (3) Å	Cell parameters from 4703 reflections
*b* = 12.121 (3) Å	θ = 3.2–27.5°
*c* = 12.295 (3) Å	µ = 0.27 mm^−^^1^
α = 101.75°	*T* = 93 K
β = 111.843 (3)°	Block, colourless
γ = 110.833 (2)°	0.40 × 0.33 × 0.30 mm
*V* = 1435.1 (6) Å^3^	

### Data collection

**Table:**

Rigaku SPIDER diffractometer	5120 reflections with *I* > 2σ(*I*)
Radiation source: Rotating Anode	*R*_int_ = 0.021
graphite	θ_max_ = 27.5°, θ_min_ = 3.2°
ω scans	*h* = −14→14
11770 measured reflections	*k* = −15→15
6317 independent reflections	*l* = −15→15

### Refinement

**Table:**

Refinement on *F*^2^	Primary atom site location: structure-invariant direct methods
Least-squares matrix: full	Secondary atom site location: difference Fourier map
*R*[*F*^2^ > 2σ(*F*^2^)] = 0.036	Hydrogen site location: inferred from neighbouring sites
*wR*(*F*^2^) = 0.095	H atoms treated by a mixture of independent and constrained refinement
*S* = 1.00	*w* = 1/[σ^2^(*F*_o_^2^) + (0.0495*P*)^2^ + 0.26*P*] where *P* = (*F*_o_^2^ + 2*F*_c_^2^)/3
6317 reflections	(Δ/σ)_max_ = 0.001
373 parameters	Δρ_max_ = 0.35 e Å^−^^3^
0 restraints	Δρ_min_ = −0.21 e Å^−^^3^

### Special details

**Table:**

Geometry. All e.s.d.'s (except the e.s.d. in the dihedral angle between two l.s. planes) are estimated using the full covariance matrix. The cell e.s.d.'s are taken into account individually in the estimation of e.s.d.'s in distances, angles and torsion angles; correlations between e.s.d.'s in cell parameters are only used when they are defined by crystal symmetry. An approximate (isotropic) treatment of cell e.s.d.'s is used for estimating e.s.d.'s involving l.s. planes.
Refinement. Refinement of *F*^2^ against ALL reflections. The weighted *R*-factor *wR* and goodness of fit *S* are based on *F*^2^, conventional *R*-factors *R* are based on *F*, with *F* set to zero for negative *F*^2^. The threshold expression of *F*^2^ > σ(*F*^2^) is used only for calculating *R*-factors(gt) *etc*. and is not relevant to the choice of reflections for refinement. *R*-factors based on *F*^2^ are statistically about twice as large as those based on *F*, and *R*- factors based on ALL data will be even larger.

### Fractional atomic coordinates and isotropic or equivalent isotropic displacement parameters (Å^2^)

**Table:**

	*x*	*y*	*z*	*U*_iso_*/*U*_eq_	
Cl1	1.14954 (4)	0.24083 (4)	1.06116 (4)	0.02618 (10)	
Cl2	0.34115 (4)	1.10782 (4)	0.16440 (4)	0.02949 (11)	
O1	0.72486 (10)	0.35489 (10)	0.60000 (10)	0.0254 (2)	
O2	0.78897 (10)	0.97633 (10)	0.58937 (10)	0.0223 (2)	
N1	0.93824 (12)	0.51036 (12)	0.75515 (11)	0.0166 (3)	
N2	0.58949 (12)	0.82237 (12)	0.41155 (11)	0.0185 (3)	
C1	0.79112 (14)	0.56799 (13)	0.61081 (12)	0.0152 (3)	
C2	0.68071 (14)	0.57861 (13)	0.60897 (13)	0.0175 (3)	
H2	0.6230	0.5214	0.6305	0.021*	
C3	0.65240 (14)	0.67394 (13)	0.57528 (13)	0.0172 (3)	
H3	0.5759	0.6806	0.5746	0.021*	
C4	0.73406 (14)	0.75669 (13)	0.54364 (12)	0.0158 (3)	
C5	0.92703 (15)	0.82315 (14)	0.49705 (14)	0.0204 (3)	
H5	0.9085	0.8876	0.4746	0.024*	
C6	1.03101 (16)	0.80760 (15)	0.48905 (15)	0.0239 (3)	
H6	1.0837	0.8606	0.4607	0.029*	
C7	1.06006 (16)	0.71245 (15)	0.52307 (14)	0.0232 (3)	
H7	1.1325	0.7018	0.5176	0.028*	
C8	0.98458 (15)	0.63599 (14)	0.56367 (13)	0.0193 (3)	
H8	1.0060	0.5731	0.5869	0.023*	
C9	0.87465 (14)	0.64829 (13)	0.57208 (12)	0.0151 (3)	
C10	0.84593 (14)	0.74473 (13)	0.53832 (12)	0.0157 (3)	
C11	0.81469 (14)	0.46640 (13)	0.65323 (13)	0.0159 (3)	
C12	0.98484 (14)	0.44042 (13)	0.82256 (13)	0.0153 (3)	
C13	0.91798 (15)	0.30732 (13)	0.77869 (13)	0.0187 (3)	
H13	0.8375	0.2585	0.6987	0.022*	
C14	0.96930 (15)	0.24596 (14)	0.85217 (14)	0.0203 (3)	
H14	0.9242	0.1552	0.8226	0.024*	
C15	1.08613 (15)	0.31781 (14)	0.96829 (13)	0.0181 (3)	
C16	1.15477 (14)	0.45050 (14)	1.01369 (13)	0.0183 (3)	
H16	1.2352	0.4988	1.0937	0.022*	
C17	1.10346 (14)	0.51115 (14)	0.93975 (13)	0.0177 (3)	
H17	1.1496	0.6019	0.9693	0.021*	
C18	0.70864 (14)	0.86425 (13)	0.51829 (13)	0.0167 (3)	
C19	0.53522 (14)	0.89601 (13)	0.35801 (13)	0.0164 (3)	
C20	0.58492 (14)	1.02652 (13)	0.42169 (14)	0.0180 (3)	
H20	0.6596	1.0709	0.5056	0.022*	
C21	0.52446 (15)	1.09143 (14)	0.36147 (13)	0.0190 (3)	
H21	0.5579	1.1804	0.4038	0.023*	
C22	0.41559 (15)	1.02545 (14)	0.23974 (13)	0.0184 (3)	
C23	0.36372 (15)	0.89561 (14)	0.17557 (14)	0.0211 (3)	
H23	0.2882	0.8515	0.0921	0.025*	
C24	0.42413 (15)	0.83144 (14)	0.23555 (14)	0.0209 (3)	
H24	0.3895	0.7423	0.1928	0.025*	
O3	0.48112 (11)	0.56240 (10)	0.23871 (10)	0.0235 (2)	
O4	0.12745 (11)	0.77715 (10)	−0.12739 (10)	0.0301 (3)	
N3	0.62413 (14)	0.49392 (13)	0.20677 (14)	0.0286 (3)	
N4	0.19877 (13)	0.95703 (12)	−0.16672 (12)	0.0244 (3)	
C25	0.53141 (16)	0.48995 (15)	0.24215 (15)	0.0254 (3)	
H25	0.5014	0.4255	0.2728	0.030*	
C26	0.6798 (2)	0.4056 (2)	0.2165 (2)	0.0498 (5)	
H26A	0.6433	0.3516	0.2570	0.060*	
H26B	0.7790	0.4541	0.2676	0.060*	
H26C	0.6544	0.3516	0.1313	0.060*	
C27	0.6761 (2)	0.58983 (19)	0.1596 (2)	0.0437 (5)	
H27A	0.6471	0.5480	0.0697	0.052*	
H27B	0.7756	0.6359	0.2084	0.052*	
H27C	0.6406	0.6504	0.1690	0.052*	
C28	0.11021 (16)	0.86429 (15)	−0.15544 (14)	0.0246 (3)	
H28	0.0271	0.8645	−0.1699	0.030*	
C29	0.1742 (2)	1.05877 (17)	−0.19713 (17)	0.0386 (4)	
H29A	0.0801	1.0386	−0.2202	0.046*	
H29B	0.1897	1.0665	−0.2687	0.046*	
H29C	0.2363	1.1398	−0.1230	0.046*	
C30	0.32963 (19)	0.96522 (19)	−0.14314 (19)	0.0411 (5)	
H30A	0.4025	1.0433	−0.0689	0.049*	
H30B	0.3412	0.9675	−0.2176	0.049*	
H30C	0.3334	0.8903	−0.1271	0.049*	
H1N	0.9893 (17)	0.5908 (17)	0.7908 (16)	0.024 (4)*	
H2N	0.5477 (18)	0.7389 (19)	0.3633 (18)	0.037 (5)*	

### Atomic displacement parameters (Å^2^)

**Table:**

	*U*^11^	*U*^22^	*U*^33^	*U*^12^	*U*^13^	*U*^23^
Cl1	0.0315 (2)	0.0296 (2)	0.02474 (19)	0.02073 (17)	0.01110 (17)	0.01733 (16)
Cl2	0.0385 (2)	0.0270 (2)	0.02280 (19)	0.02399 (18)	0.00642 (17)	0.01041 (15)
O1	0.0206 (5)	0.0156 (5)	0.0267 (6)	0.0062 (4)	0.0015 (5)	0.0073 (4)
O2	0.0198 (5)	0.0163 (5)	0.0216 (5)	0.0068 (4)	0.0036 (4)	0.0067 (4)
N1	0.0161 (6)	0.0126 (6)	0.0166 (6)	0.0056 (5)	0.0048 (5)	0.0057 (5)
N2	0.0186 (6)	0.0138 (6)	0.0178 (6)	0.0082 (5)	0.0034 (5)	0.0058 (5)
C1	0.0171 (7)	0.0143 (6)	0.0116 (6)	0.0072 (5)	0.0048 (6)	0.0049 (5)
C2	0.0183 (7)	0.0165 (7)	0.0167 (7)	0.0069 (6)	0.0082 (6)	0.0077 (5)
C3	0.0153 (7)	0.0188 (7)	0.0176 (7)	0.0090 (6)	0.0073 (6)	0.0074 (6)
C4	0.0164 (7)	0.0159 (7)	0.0120 (6)	0.0081 (6)	0.0040 (6)	0.0049 (5)
C5	0.0239 (8)	0.0190 (7)	0.0210 (7)	0.0105 (6)	0.0113 (6)	0.0115 (6)
C6	0.0274 (8)	0.0242 (8)	0.0264 (8)	0.0112 (7)	0.0182 (7)	0.0137 (6)
C7	0.0239 (8)	0.0254 (8)	0.0263 (8)	0.0137 (7)	0.0160 (7)	0.0099 (6)
C8	0.0232 (8)	0.0198 (7)	0.0183 (7)	0.0125 (6)	0.0108 (6)	0.0080 (6)
C9	0.0161 (7)	0.0149 (6)	0.0111 (6)	0.0067 (5)	0.0049 (6)	0.0037 (5)
C10	0.0171 (7)	0.0155 (7)	0.0124 (6)	0.0072 (6)	0.0057 (6)	0.0056 (5)
C11	0.0175 (7)	0.0177 (7)	0.0142 (6)	0.0093 (6)	0.0079 (6)	0.0073 (5)
C12	0.0179 (7)	0.0175 (7)	0.0159 (6)	0.0104 (6)	0.0101 (6)	0.0091 (5)
C13	0.0187 (7)	0.0167 (7)	0.0158 (7)	0.0074 (6)	0.0052 (6)	0.0055 (5)
C14	0.0230 (8)	0.0175 (7)	0.0215 (7)	0.0107 (6)	0.0101 (6)	0.0089 (6)
C15	0.0206 (7)	0.0247 (8)	0.0196 (7)	0.0160 (6)	0.0119 (6)	0.0141 (6)
C16	0.0170 (7)	0.0231 (7)	0.0157 (7)	0.0109 (6)	0.0073 (6)	0.0077 (6)
C17	0.0169 (7)	0.0161 (7)	0.0189 (7)	0.0076 (6)	0.0077 (6)	0.0067 (5)
C18	0.0168 (7)	0.0188 (7)	0.0167 (7)	0.0088 (6)	0.0087 (6)	0.0090 (5)
C19	0.0155 (7)	0.0169 (7)	0.0187 (7)	0.0086 (6)	0.0081 (6)	0.0092 (6)
C20	0.0180 (7)	0.0175 (7)	0.0169 (7)	0.0088 (6)	0.0071 (6)	0.0057 (5)
C21	0.0225 (8)	0.0170 (7)	0.0197 (7)	0.0118 (6)	0.0102 (6)	0.0069 (6)
C22	0.0207 (7)	0.0212 (7)	0.0193 (7)	0.0147 (6)	0.0092 (6)	0.0108 (6)
C23	0.0181 (7)	0.0204 (7)	0.0184 (7)	0.0093 (6)	0.0032 (6)	0.0067 (6)
C24	0.0208 (8)	0.0141 (7)	0.0209 (7)	0.0076 (6)	0.0048 (6)	0.0056 (6)
O3	0.0238 (6)	0.0207 (5)	0.0253 (6)	0.0115 (5)	0.0114 (5)	0.0072 (4)
O4	0.0312 (6)	0.0197 (6)	0.0207 (5)	0.0045 (5)	0.0014 (5)	0.0093 (4)
N3	0.0289 (8)	0.0274 (7)	0.0382 (8)	0.0157 (6)	0.0219 (7)	0.0135 (6)
N4	0.0322 (7)	0.0215 (7)	0.0198 (6)	0.0118 (6)	0.0130 (6)	0.0095 (5)
C25	0.0294 (9)	0.0227 (8)	0.0293 (8)	0.0133 (7)	0.0175 (7)	0.0117 (7)
C26	0.0552 (13)	0.0498 (13)	0.0763 (16)	0.0392 (11)	0.0446 (12)	0.0316 (12)
C27	0.0473 (12)	0.0422 (11)	0.0649 (14)	0.0232 (9)	0.0431 (11)	0.0289 (10)
C28	0.0267 (8)	0.0238 (8)	0.0165 (7)	0.0089 (7)	0.0070 (6)	0.0077 (6)
C29	0.0612 (12)	0.0243 (9)	0.0314 (9)	0.0197 (9)	0.0220 (9)	0.0151 (7)
C30	0.0397 (11)	0.0399 (11)	0.0466 (11)	0.0152 (9)	0.0266 (10)	0.0176 (9)

### Geometric parameters (Å, °)

**Table:**

Cl1—C15	1.7462 (14)	C16—C17	1.3877 (19)
Cl2—C22	1.7473 (14)	C16—H16	0.95
O1—C11	1.2240 (17)	C17—H17	0.95
O2—C18	1.2238 (17)	C19—C24	1.395 (2)
N1—C11	1.3580 (18)	C19—C20	1.3954 (19)
N1—C12	1.4138 (17)	C20—C21	1.3930 (19)
N1—H1N	0.849 (18)	C20—H20	0.95
N2—C18	1.3594 (18)	C21—C22	1.380 (2)
N2—C19	1.4110 (17)	C21—H21	0.95
N2—H2N	0.895 (19)	C22—C23	1.385 (2)
C1—C2	1.373 (2)	C23—C24	1.3837 (19)
C1—C9	1.4264 (19)	C23—H23	0.95
C1—C11	1.5062 (18)	C24—H24	0.95
C2—C3	1.4117 (19)	O3—C25	1.2304 (18)
C2—H2	0.95	O4—C28	1.2363 (19)
C3—C4	1.366 (2)	N3—C25	1.330 (2)
C3—H3	0.95	N3—C27	1.451 (2)
C4—C10	1.428 (2)	N3—C26	1.453 (2)
C4—C18	1.5062 (19)	N4—C28	1.319 (2)
C5—C6	1.366 (2)	N4—C29	1.454 (2)
C5—C10	1.421 (2)	N4—C30	1.454 (2)
C5—H5	0.95	C25—H25	0.95
C6—C7	1.415 (2)	C26—H26A	0.98
C6—H6	0.95	C26—H26B	0.98
C7—C8	1.364 (2)	C26—H26C	0.98
C7—H7	0.95	C27—H27A	0.98
C8—C9	1.421 (2)	C27—H27B	0.98
C8—H8	0.95	C27—H27C	0.98
C9—C10	1.4271 (19)	C28—H28	0.95
C12—C13	1.3930 (19)	C29—H29A	0.98
C12—C17	1.3949 (19)	C29—H29B	0.98
C13—C14	1.3914 (19)	C29—H29C	0.98
C13—H13	0.95	C30—H30A	0.98
C14—C15	1.380 (2)	C30—H30B	0.98
C14—H14	0.95	C30—H30C	0.98
C15—C16	1.388 (2)		
			
C11—N1—C12	127.53 (12)	O2—C18—N2	125.43 (13)
C11—N1—H1N	117.1 (11)	O2—C18—C4	121.68 (12)
C12—N1—H1N	114.5 (12)	N2—C18—C4	112.89 (12)
C18—N2—C19	128.18 (12)	C24—C19—C20	119.68 (13)
C18—N2—H2N	115.0 (12)	C24—C19—N2	116.70 (12)
C19—N2—H2N	116.0 (12)	C20—C19—N2	123.61 (13)
C2—C1—C9	120.61 (13)	C21—C20—C19	119.64 (13)
C2—C1—C11	115.86 (12)	C21—C20—H20	120.2
C9—C1—C11	123.53 (12)	C19—C20—H20	120.2
C1—C2—C3	120.43 (13)	C22—C21—C20	119.46 (13)
C1—C2—H2	119.8	C22—C21—H21	120.3
C3—C2—H2	119.8	C20—C21—H21	120.3
C4—C3—C2	120.57 (13)	C21—C22—C23	121.76 (13)
C4—C3—H3	119.7	C21—C22—Cl2	119.18 (11)
C2—C3—H3	119.7	C23—C22—Cl2	119.05 (11)
C3—C4—C10	120.67 (13)	C24—C23—C22	118.64 (13)
C3—C4—C18	119.65 (12)	C24—C23—H23	120.7
C10—C4—C18	119.65 (12)	C22—C23—H23	120.7
C6—C5—C10	121.27 (14)	C23—C24—C19	120.81 (13)
C6—C5—H5	119.4	C23—C24—H24	119.6
C10—C5—H5	119.4	C19—C24—H24	119.6
C5—C6—C7	119.86 (14)	C25—N3—C27	120.68 (14)
C5—C6—H6	120.1	C25—N3—C26	121.89 (15)
C7—C6—H6	120.1	C27—N3—C26	117.41 (15)
C8—C7—C6	120.34 (14)	C28—N4—C29	122.37 (15)
C8—C7—H7	119.8	C28—N4—C30	120.96 (14)
C6—C7—H7	119.8	C29—N4—C30	116.61 (15)
C7—C8—C9	121.50 (14)	O3—C25—N3	125.73 (16)
C7—C8—H8	119.2	O3—C25—H25	117.1
C9—C8—H8	119.2	N3—C25—H25	117.1
C8—C9—C1	123.18 (13)	N3—C26—H26A	109.5
C8—C9—C10	118.07 (13)	N3—C26—H26B	109.5
C1—C9—C10	118.74 (12)	H26A—C26—H26B	109.5
C5—C10—C9	118.95 (13)	N3—C26—H26C	109.5
C5—C10—C4	122.19 (13)	H26A—C26—H26C	109.5
C9—C10—C4	118.83 (13)	H26B—C26—H26C	109.5
O1—C11—N1	124.73 (13)	N3—C27—H27A	109.5
O1—C11—C1	120.55 (12)	N3—C27—H27B	109.5
N1—C11—C1	114.67 (12)	H27A—C27—H27B	109.5
C13—C12—C17	119.60 (13)	N3—C27—H27C	109.5
C13—C12—N1	123.54 (13)	H27A—C27—H27C	109.5
C17—C12—N1	116.86 (12)	H27B—C27—H27C	109.5
C14—C13—C12	119.91 (13)	O4—C28—N4	124.81 (16)
C14—C13—H13	120.0	O4—C28—H28	117.6
C12—C13—H13	120.0	N4—C28—H28	117.6
C15—C14—C13	119.55 (14)	N4—C29—H29A	109.5
C15—C14—H14	120.2	N4—C29—H29B	109.5
C13—C14—H14	120.2	H29A—C29—H29B	109.5
C14—C15—C16	121.55 (13)	N4—C29—H29C	109.5
C14—C15—Cl1	119.53 (11)	H29A—C29—H29C	109.5
C16—C15—Cl1	118.92 (11)	H29B—C29—H29C	109.5
C17—C16—C15	118.65 (13)	N4—C30—H30A	109.5
C17—C16—H16	120.7	N4—C30—H30B	109.5
C15—C16—H16	120.7	H30A—C30—H30B	109.5
C16—C17—C12	120.75 (13)	N4—C30—H30C	109.5
C16—C17—H17	119.6	H30A—C30—H30C	109.5
C12—C17—H17	119.6	H30B—C30—H30C	109.5
			
C9—C1—C2—C3	−3.5 (2)	C17—C12—C13—C14	0.4 (2)
C11—C1—C2—C3	177.63 (12)	N1—C12—C13—C14	−178.74 (13)
C1—C2—C3—C4	0.2 (2)	C12—C13—C14—C15	0.1 (2)
C2—C3—C4—C10	2.9 (2)	C13—C14—C15—C16	−0.4 (2)
C2—C3—C4—C18	−175.08 (12)	C13—C14—C15—Cl1	179.37 (11)
C10—C5—C6—C7	−0.4 (2)	C14—C15—C16—C17	0.1 (2)
C5—C6—C7—C8	0.1 (2)	Cl1—C15—C16—C17	−179.65 (10)
C6—C7—C8—C9	0.6 (2)	C15—C16—C17—C12	0.4 (2)
C7—C8—C9—C1	178.28 (14)	C13—C12—C17—C16	−0.7 (2)
C7—C8—C9—C10	−0.8 (2)	N1—C12—C17—C16	178.50 (13)
C2—C1—C9—C8	−175.58 (13)	C19—N2—C18—O2	2.1 (2)
C11—C1—C9—C8	3.2 (2)	C19—N2—C18—C4	−178.44 (13)
C2—C1—C9—C10	3.53 (19)	C3—C4—C18—O2	112.28 (16)
C11—C1—C9—C10	−177.64 (12)	C10—C4—C18—O2	−65.73 (18)
C6—C5—C10—C9	0.1 (2)	C3—C4—C18—N2	−67.17 (17)
C6—C5—C10—C4	−178.02 (13)	C10—C4—C18—N2	114.83 (14)
C8—C9—C10—C5	0.50 (19)	C18—N2—C19—C24	168.17 (14)
C1—C9—C10—C5	−178.66 (12)	C18—N2—C19—C20	−13.1 (2)
C8—C9—C10—C4	178.70 (12)	C24—C19—C20—C21	−1.0 (2)
C1—C9—C10—C4	−0.46 (19)	N2—C19—C20—C21	−179.70 (13)
C3—C4—C10—C5	175.41 (13)	C19—C20—C21—C22	0.4 (2)
C18—C4—C10—C5	−6.6 (2)	C20—C21—C22—C23	0.4 (2)
C3—C4—C10—C9	−2.72 (19)	C20—C21—C22—Cl2	−179.51 (11)
C18—C4—C10—C9	175.26 (12)	C21—C22—C23—C24	−0.6 (2)
C12—N1—C11—O1	−3.3 (2)	Cl2—C22—C23—C24	179.40 (12)
C12—N1—C11—C1	174.14 (12)	C22—C23—C24—C19	−0.2 (2)
C2—C1—C11—O1	55.39 (18)	C20—C19—C24—C23	0.9 (2)
C9—C1—C11—O1	−123.49 (16)	N2—C19—C24—C23	179.69 (13)
C2—C1—C11—N1	−122.19 (14)	C27—N3—C25—O3	0.4 (3)
C9—C1—C11—N1	58.93 (17)	C26—N3—C25—O3	178.82 (17)
C11—N1—C12—C13	12.7 (2)	C29—N4—C28—O4	−178.55 (14)
C11—N1—C12—C17	−166.47 (13)	C30—N4—C28—O4	−1.5 (2)

### Hydrogen-bond geometry (Å, °)

**Table:**

*D*—H···*A*	*D*—H	H···*A*	*D*···*A*	*D*—H···*A*
N2—H2N···O3	0.89 (2)	2.02 (2)	2.9040 (17)	168 (2)
C23—H23···O4	0.95	2.39	3.3258 (19)	167
N1—H1N···O4^i^	0.85 (2)	2.01 (2)	2.8536 (17)	173 (2)
C2—H2···O3^ii^	0.95	2.54	3.4458 (19)	160
C6—H6···O2^iii^	0.95	2.38	3.3214 (19)	169
C16—H16···O3^i^	0.95	2.54	3.3949 (19)	151
C21—H21···O1^iv^	0.95	2.37	3.1719 (19)	142

Symmetry codes: (i) *x*+1, *y*, *z*+1; (ii) −*x*+1, −*y*+1, −*z*+1; (iii) −*x*+2, −*y*+2, −*z*+1; (iv) *x*, *y*+1, *z*.
